# Retrospective motion correction in foetal MRI for clinical applications: existing methods, applications and integration into clinical practice

**DOI:** 10.1259/bjr.20220071

**Published:** 2022-08-08

**Authors:** Alena U. Uus, Alexia Egloff Collado, Thomas A. Roberts, Joseph V. Hajnal, Mary A. Rutherford, Maria Deprez

**Affiliations:** 1 Department of Biomedical Engineering, School Biomedical Engineering and Imaging Sciences, King’s College London, St. Thomas’ Hospital, London, United Kingdom; 2 Centre for the Developing Brain, School Biomedical Engineering and Imaging Sciences, King’s College London, St. Thomas’ Hospital, London, United Kingdom; 3 Clinical Scientific Computing, Guy’s and St Thomas’ NHS Foundation Trust, London, United Kingdom

## Abstract

Foetal MRI is a complementary imaging method to antenatal ultrasound. It provides advanced information for detection and characterisation of foetal brain and body anomalies. Even though modern single shot sequences allow fast acquisition of 2D slices with high in-plane image quality, foetal MRI is intrinsically corrupted by motion. Foetal motion leads to loss of structural continuity and corrupted 3D volumetric information in stacks of slices. Furthermore, the arbitrary and constantly changing position of the foetus requires dynamic readjustment of acquisition planes during scanning.

Over the past 15 years, a series of works have addressed this challenge via retrospective motion correction performed in the image domain using slice-to-volume registration (SVR). SVR-based methods allow reconstruction of 3D isotropic high-resolution images of the foetal brain and body from multiple motion-corrupted stacks. 3D reconstructed images can be reoriented in any plane and used for volume rendering for advanced visualisation. This also potentially reduces the errors in biometry and organ volumetry which are conventionally performed using 2D image data.

The reported early-stage results on the added value of SVR reconstruction are promising and 3D foetal MRI is gradually evolving towards clinical validation. However, the next step on the way to full integration into clinical practice would require extensive evaluation and standardisation of the different components of SVR reconstruction methods and further optimisation of existing software solutions for large-scale application.

## Introduction: Motion in Foetal MRI

MRI provides complementary information to antenatal ultrasound in the evaluation of the high-risk foetuses and is now an established clinical technique to confirm or further characterise foetal anomalies.^1–3^ Different studies have proven the added benefit of MRI for diagnosis of both central nervous system (CNS) and non-CNS abnormalities for foetal and post-natal management and parental counselling. While image quality is hampered by inevitable foetal motion and maternal breathing, the development of fast single shot acquisition approaches has overcome, in part, artefacts associated with motion within individual 2D slices and the need for maternal sedation. However, a conventional MRI examination still relies on repeated acquisitions to ensure not only full coverage but also precise three traditional orthogonal views of the foetus.

**Figure 1:**
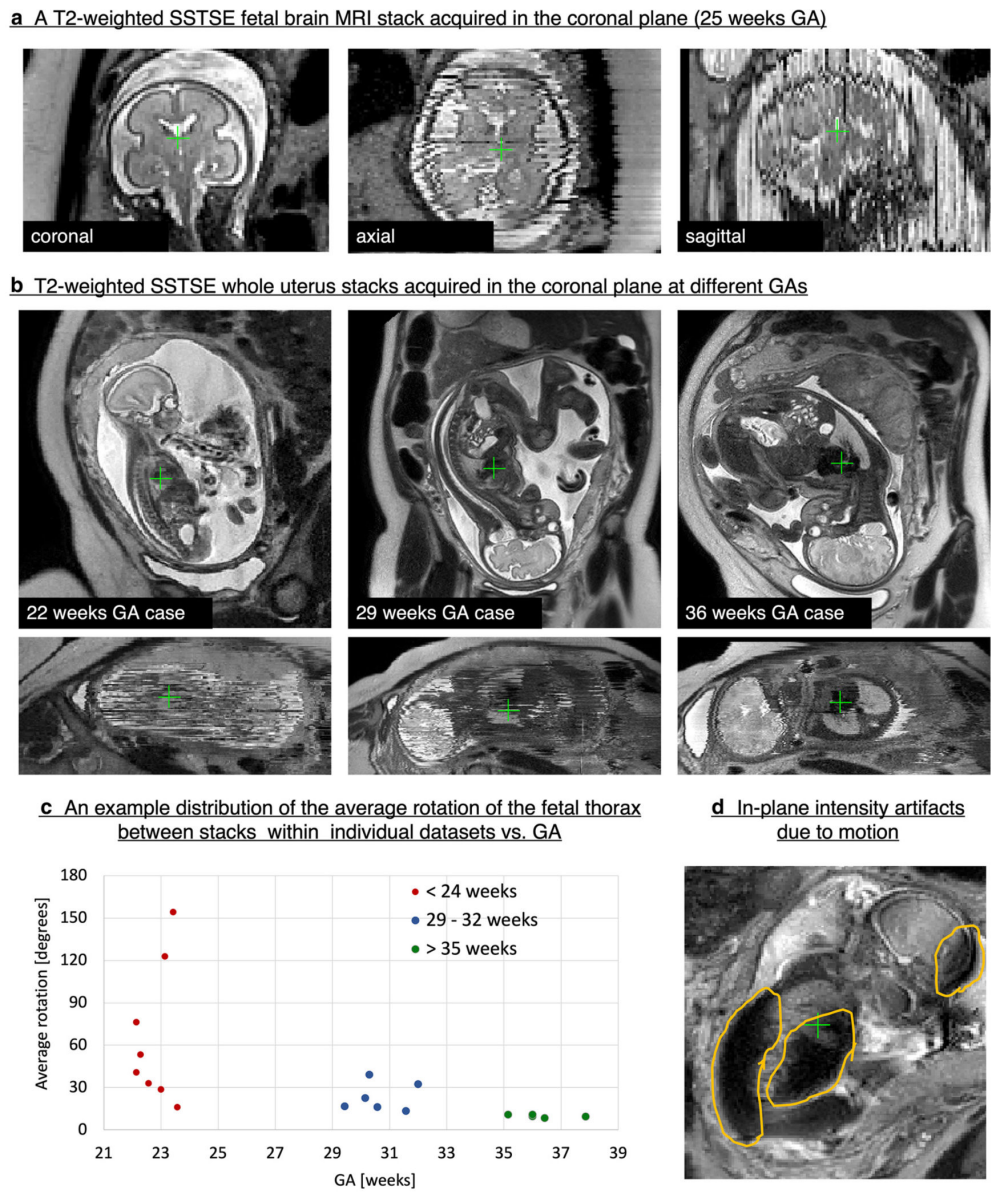
Motion in foetal MRI: examples of *T2*-weighted SSTSE stacks of 2D slices. The MRI datasets used in this example were acquired at St.Thomas’ Hospital, London.

The coronal stack of 2D slices shown in Figure 1a covers the foetal brain and has high in-plane contrast and signal-to-noise ratio (SNR). Yet, even minor motion leads to loss of structural continuity between the slices and corruption of volumetric information. Furthermore, the arbitrary and constantly changing global foetal position within the uterus (Figure 1b) requires dynamic adjustment of acquisition planes in order to capture precise orthogonal anatomic views, which is not always achievable in practical examinations.

Foetal MRI is generally performed between 20 and 38 weeks gestational age (GA). Motion is more marked in younger GA datasets due to, in part, a relatively large amniotic fluid volume and room for manoeuvre.^4^ Figure 1b shows examples of *T2*-weighted coronal whole uterus stacks acquired at 22, 29 and 36 weeks GA. As can be seen in the through-plane views, the 22 weeks case is severely motion-corrupted and motion is less prominent with increasing GA. This is also reflected in Figure 1c which demonstrates that average foetal thorax motion (rotation between stacks within individual datasets) decreases with increasing foetal age. However, the degree of motion typically varies from case to case. Some early GA foetuses do remain stationary, while head motion of term foetuses may significantly compromise image quality.

The types of foetal motion (see Supplementary Video 1) include: translations and rotations of the foetal head and trunk, bending of the spine that leads to deformation of internal organs, motion of the limbs and physiological motion (*e.g*. cardiac, pseudobreathing, swallowing). Motion artefacts also arise from maternal respiratory motion. This is particularly pronounced when imaging foetal brain in breech position. Foetal motion may also result in signal intensity artefacts in individual slices (Figure 1d) that undermine visualisation and quantification.

While the modern fast and motion resistant acquisition sequences specifically designed for foetal MRI^5^ provide image quality for individual slices, there have been no reported clinically accepted solutions for prospective motion correction in 3D. Motion correction of the foetal brain and body can also be performed retrospectively in the image domain. It is based on slice-to-volume-registration (SVR) methods for reconstruction of 3D isotropic images from multiple stacks of 2D slices, described in sections below. This approach has the potential to shorten acquisition times in terms of the number and orientation of acquired stacks and enable objective 3D-based inspection and quantification of the developing foetus.

### Acquisition protocols in foetal MRI

A common MRI protocol for foetal imaging begins with a three-plane localiser, followed by a coronal large field of view *T2*-weighted (*T2*W) single shot acquisition through the entire uterus. These initial images are used to examine the foetal lie, cervix and placenta and for planning the rest of the imaging protocol. Smaller field of view images are obtained through the foetal brain and body and include axial, sagittal and coronal planes. Due to ongoing foetal motion, perpendicular planes are individually planned after each sequence.

The two main sequences used in a foetal MRI protocol are: *T2W* half Fourier single-shot fast spin echo sequences (Figure 1a) (vendor-specific names include Philips: SSTSE; Siemens: HASTE; GE, Hitachi: SSFSE; Toshiba: FASE), which provides good anatomic detail, and steady-state free precession (Philips: Balanced FFE; Siemens: TRUEFISP; GE: FIESTA; Toshiba: True SSFP; Hitachi: Balanced SARGE) with bright blood appearance, which is used to identify cardiac, vascular and fluid-filled structures.

*T1W* spoiled gradient echo (Philips: T1-FFE; Siemens: FLASH; GE: SPGR; Hitachi: RF spoiled SARGE; Toshiba: Fast FE) images can help to detect haemorrhage and calcification, and identify meconium, fat and thyroid tissue. Echoplanar images, gradient echo, spin echo and with diffusion weighting can also be obtained for additional information.

The acquisition parameters and number of stacks used for each sequence varies depending on local guidelines, such as specific absorption rate (SAR) limitations, and the hardware available, such as magnet field strength and radiofrequency (RF) coil design. Broadly, for SSTSE sequences the optimal tissue contrast is achieved at echo times (TEs) 120–180 ms for the foetal brain^6^ and TE ≤ 120 ms for the foetal body.^7^ The choice of the number of stacks, slice thickness and slice gap depends on the total scanning time limits. Thick slices (4–5 mm) result in faster acquisition and higher SNR but can lead to insufficient sampling for small organ features. Thin slices (≤3 mm) provide higher image resolution but can result in more corruption by foetal motion due to longer scanning time (*e.g*., Figure 1b).

**Figure 2:**
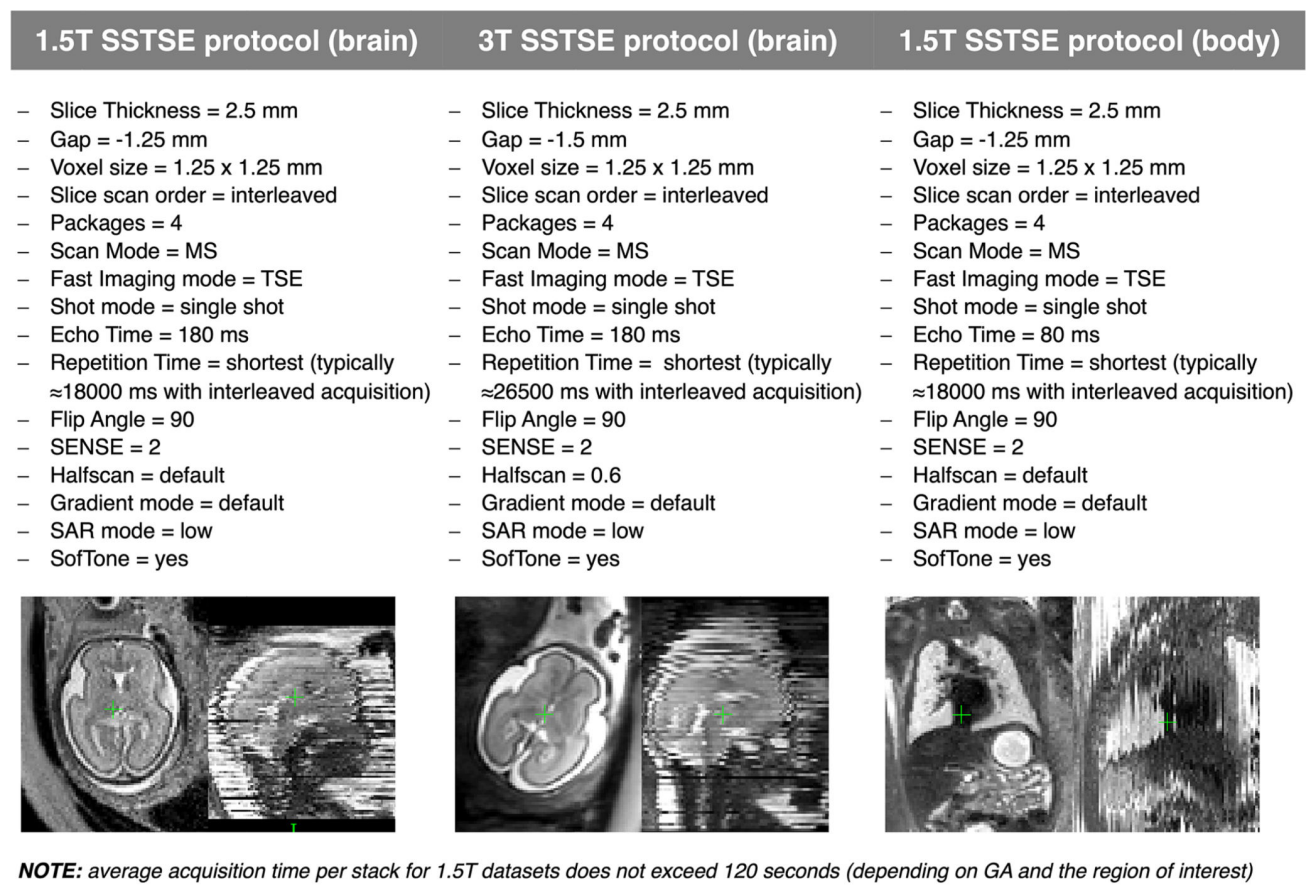
Motion-resistant *T2W* SSTSE 1.5 and 3 T protocols optimised for 3D SVR-based reconstruction at St.Thomas’ Hospital, London. GA, gestational age; SAR, specific absorption rate.

Examples of typical *T2W* SSTSE 1.5 and 3 T protocols optimised at St.Thomas’ Hospital, London for SVR reconstruction of 3D images are provided in Figure 2. In general, the average acquisition time per stack depends on the size of the scanned region (and GA of the subjects) and for 1.5 T datasets (Figure 2) does not exceed 120 s.

## Motion Correction Techniques for Foetal MRI

### Foetal brain

**Figure 3:**
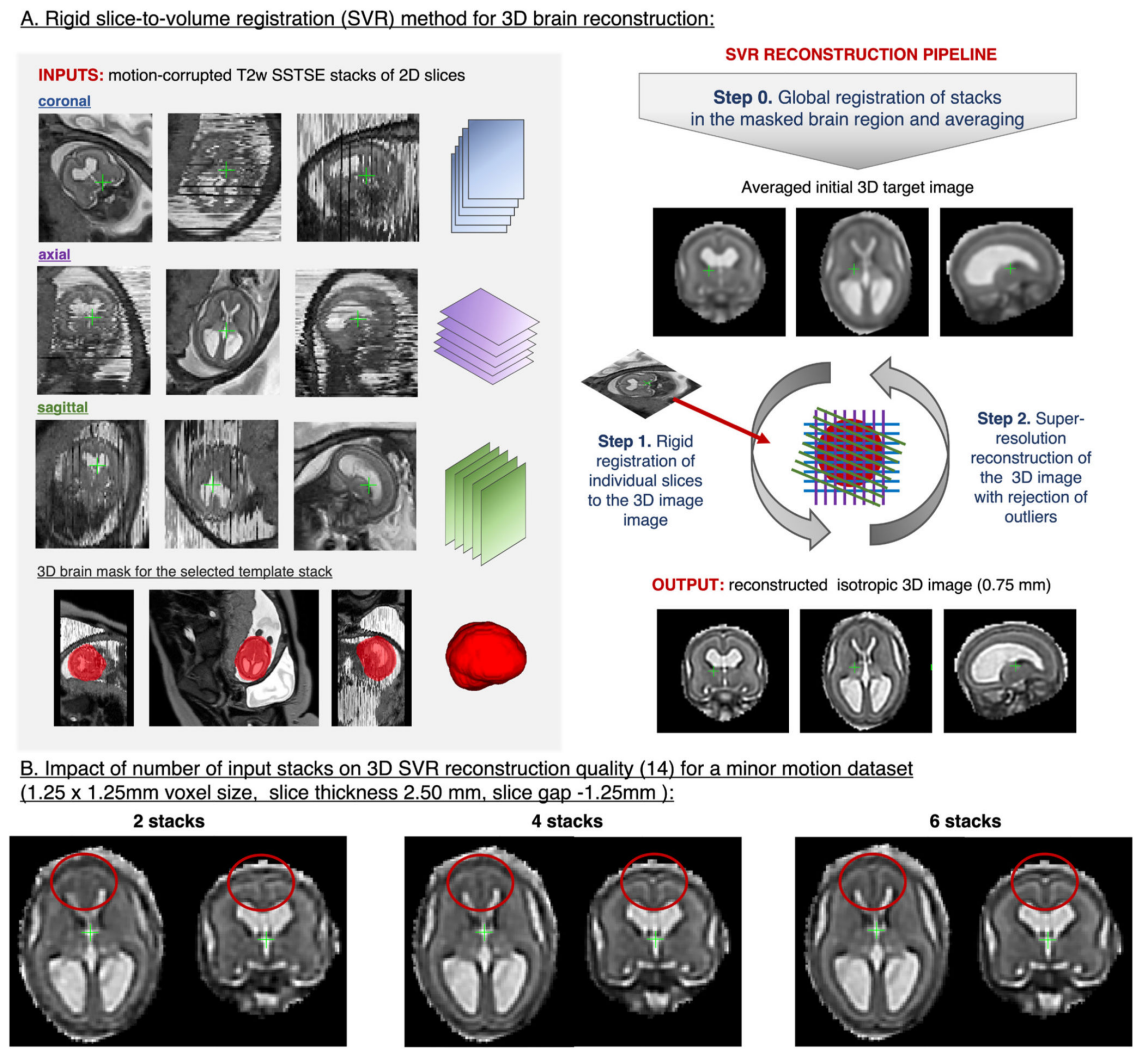
3D SVR reconstruction for foetal brain MRI. This example is based on the MRI dataset acquired at St.Thomas’ Hospital, London and reconstructed using the classical SVR. SVR, slice-to-volume registration

Despite corruption by motion, excellent visualisation of the foetal brain in 2D MRI slices (Figure 1a) brought a significant added value in diagnosis of foetal brain abnormalities.^1,8^ Clinically required stacks with multiple orthogonal views (Figure 3a.) provide ideal input data for motion-correction algorithms based on SVR.

The main steps of SVR methodology for 3D foetal brain reconstruction are illustrated in Figure 3a. At first, the input stacks are globally aligned in the masked brain region, followed by generation of an average initial 3D image (Step 0). Next, each of the 2D slices is rigidly registered to the current estimation of the 3D image (Step 1).^9–11^ This is followed by super-resolution reconstruction^12–15^ of the new 3D image from all aligned slices (Step 2). The interleaved Steps 1–2 are then repeated several times to ensure convergence to a stable solution. The method also includes rejection of misaligned or corrupted slices (*e.g*. Figure 1d).

The super-resolution technique removes the blurring introduced by relatively large slice thickness in acquired foetal MRI. Although it does not necessarily increase the acquired in-plane slice resolution (*e.g*. 1.25 × 1.25 mm), interpolation of the MRI signal during reconstruction results in high-resolution isotropic images (*e.g*. 0.75 mm). A set of examples of 3D SVR brain reconstructions (performed using the SVR method implementation from^14^) are presented in Supplementary Video 2 and Supplementary Figure 1. The quality of reconstructed 3D images is not directly affected by the field strength but depends on the amount of motion and SNR levels in the original stacks and the degree of oversampling. Thick slices (4–5 mm) add more blurring and provide sparser spatial coverage than thin slices (<3 mm) resulting in lower quality reconstructed 3D images (Supplementary Figure 2). Increasing the number of stacks (Figure 3b) generally improves reconstruction quality for small features and severe motion cases.

### Foetal body

**Figure 4:**
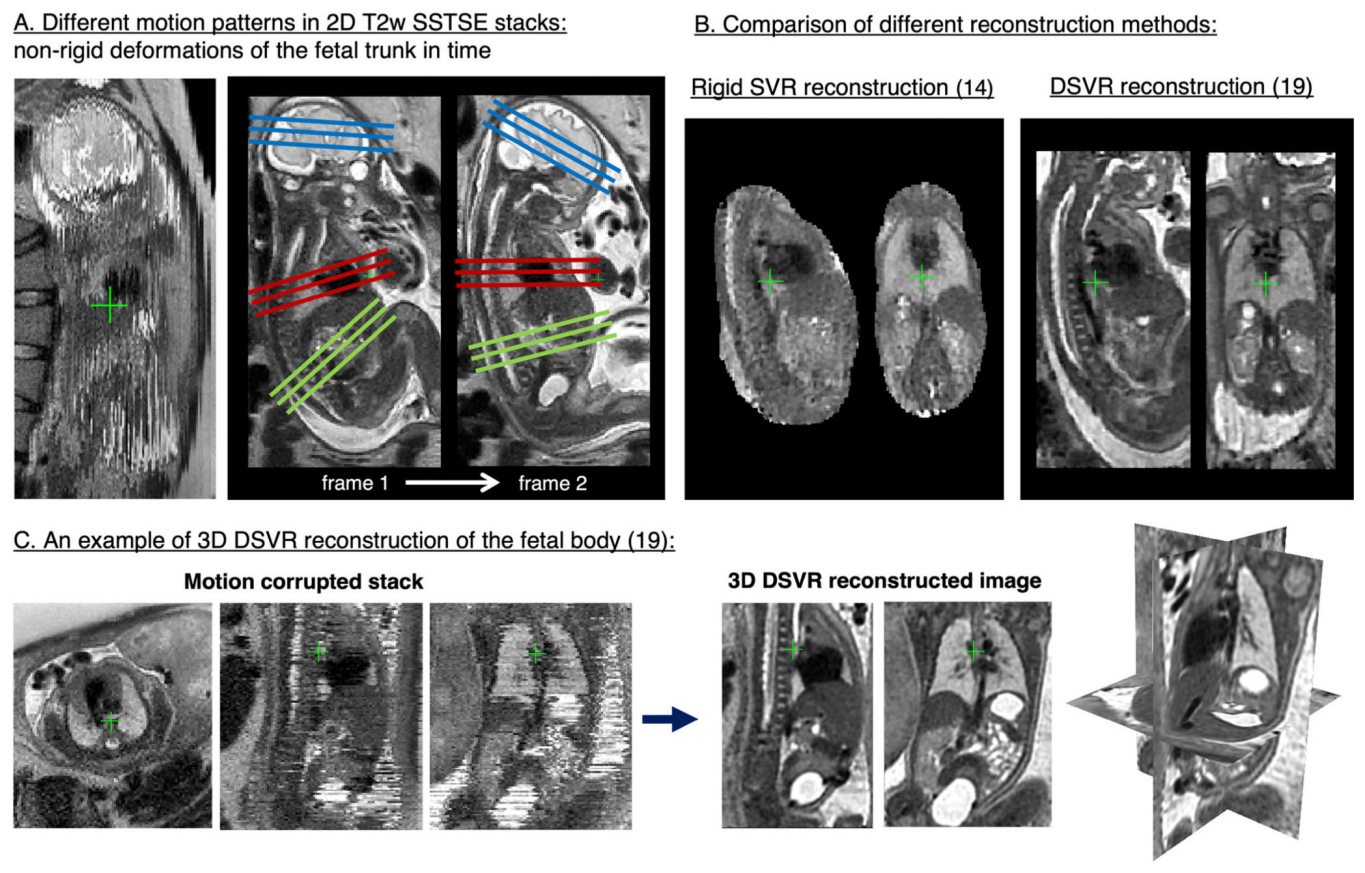
3D DSVR reconstruction for foetal body MRI. These examples are based on the MRI datasets acquired at St.Thomas’ Hospital, London. DSVR, deformable SVR; SVR, slice-to-volume registration.

More recently, SVR-based reconstruction techniques have been applied to reconstruct 3D volumes of the foetal body. The classical SVR methods based on the rigid motion model focused only on the foetal brain, since the brain (and skull) does not deform. This is however not true for the elastic foetal body organs which can deform in a non-rigid way due to bending and stretching of the spine (Figure 4a). This resulted in extension of rigid SVR techniques to correct deformation using patch-based approach.^16^

Reconstruction of foetal body organs was initially explored using the standard rigid SVR algorithm to reconstruct the foetal thorax^17^ and to visualise 3D static foetal cardiac vasculature in congenital heart disease.^18^ Incorporating correction of non-rigid deformations of the foetal body through deformable SVR (DSVR) resulted in superior image quality^19^ see Figure 4b-c. DSVR-reconstructed images provide good visualisation of various body organs in 3D, as illustrated in,^20^ Supplementary Video 3 and Supplementary Figures3 and 4.

### Artificial intelligence for 3D foetal MRI

**Figure 5:**
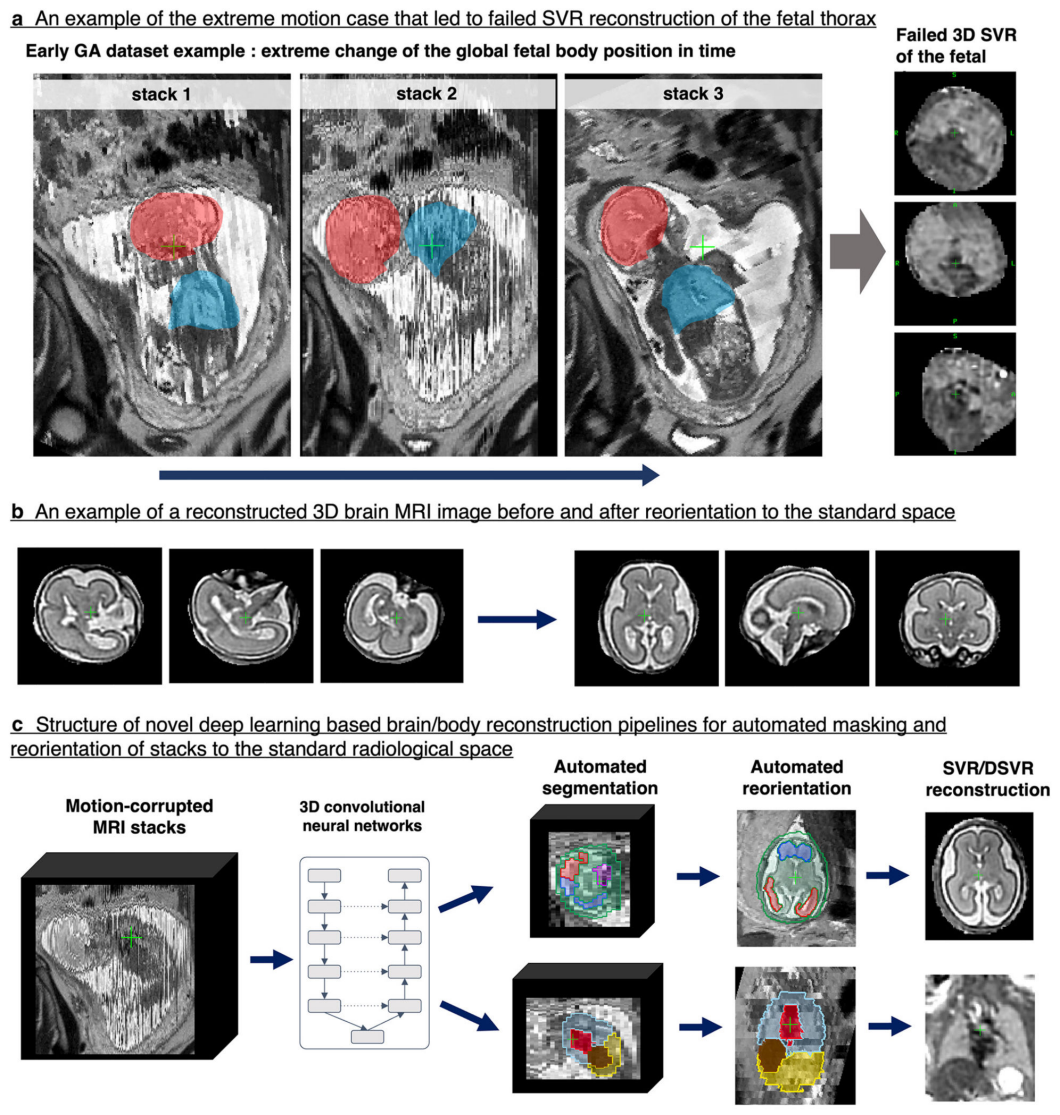
Main limitations of the classical SVR-based methods for foetal MRI and structure of AI-based solutions for automated reconstruction. This example is based on the MRI datasets acquired at St.Thomas’ Hospital, London. AI, artificial intelligence; SVR, slice-to-volume registration.

Until recently, classical SVR motion correction techniques had a number of challenges that limited their use for routine clinical applications. Firstly, extreme foetal motion can result in a reconstruction failure (*e.g*. Figure 5a) because classical registration methods cannot resolve large (>60°) rotations. This primarily affects a small proportion of early gestation (Figure 1c) and polyhydramnios cases. Secondly, classical SVR (Figure 2A) and DSVR pipelines require a brain or body mask manually drawn on the input stacks (using software tools such as ITK-SNAP^21^), manual selection of stacks, and manual reorientation of the output 3D image to the standard radiological plane (Figure 5b). These manual tasks are time consuming and operator-dependent which can cause inefficient and inconsistent clinical workflows.

Recent advances in artificial intelligence (AI) offer tractable solutions to these problems. AI techniques have proven extremely successful in automated localisation and segmentation of the foetal brain.^22,23^ Automated reorientation of individual brain slices as well as 3D volumes into standard orientation has also been achieved.^24,25^ These techniques allow fully automated correction of excessive foetal motion and reorientation of the reconstructed volume into to standard planes. This work was also extended to deformable motion-correction and reconstruction of the foetal body^26^ (Figure 5c). The major advantage of these automation steps is that they allow for efficient, routine use and unbiased reconstruction of multiple foetuses for large-scale studies. Whereas previously a standard SVR reconstruction with required manual input may take upwards of 30 min (when factoring in manual intervention in addition to computational time) it is now possible to automatically reconstruct tens or hundreds of cases per day without user input. However, the current automated methods are limited in the presence of multiple foetal brains/bodies in multiple gestation MRI scans, and this has not been addressed yet.

## Advantages of Motion-Corrected Foetal MRI for Clinical Practice

### Scanning time

In general, abnormalities of the foetal brain and body can be correctly diagnosed from 2D MRI, however, this requires accurate manual positioning of the acquisition planes in the three standard orientations. As the foetus moves, the brain can change orientation during the scan, and repeat acquisitions are often required. Instead, with SVR methods the resultant motion-corrected volumes can be reorientated to depict any desired plane through the brain. This is advantageous because image planning is less stringent for the scanner operator and there is less need to repeat scans. Consequently, this potentially results in a more efficient scanning protocol and a significant reduction of the total scanning time.

### Diagnosis and prognosis

**Figure 6:**
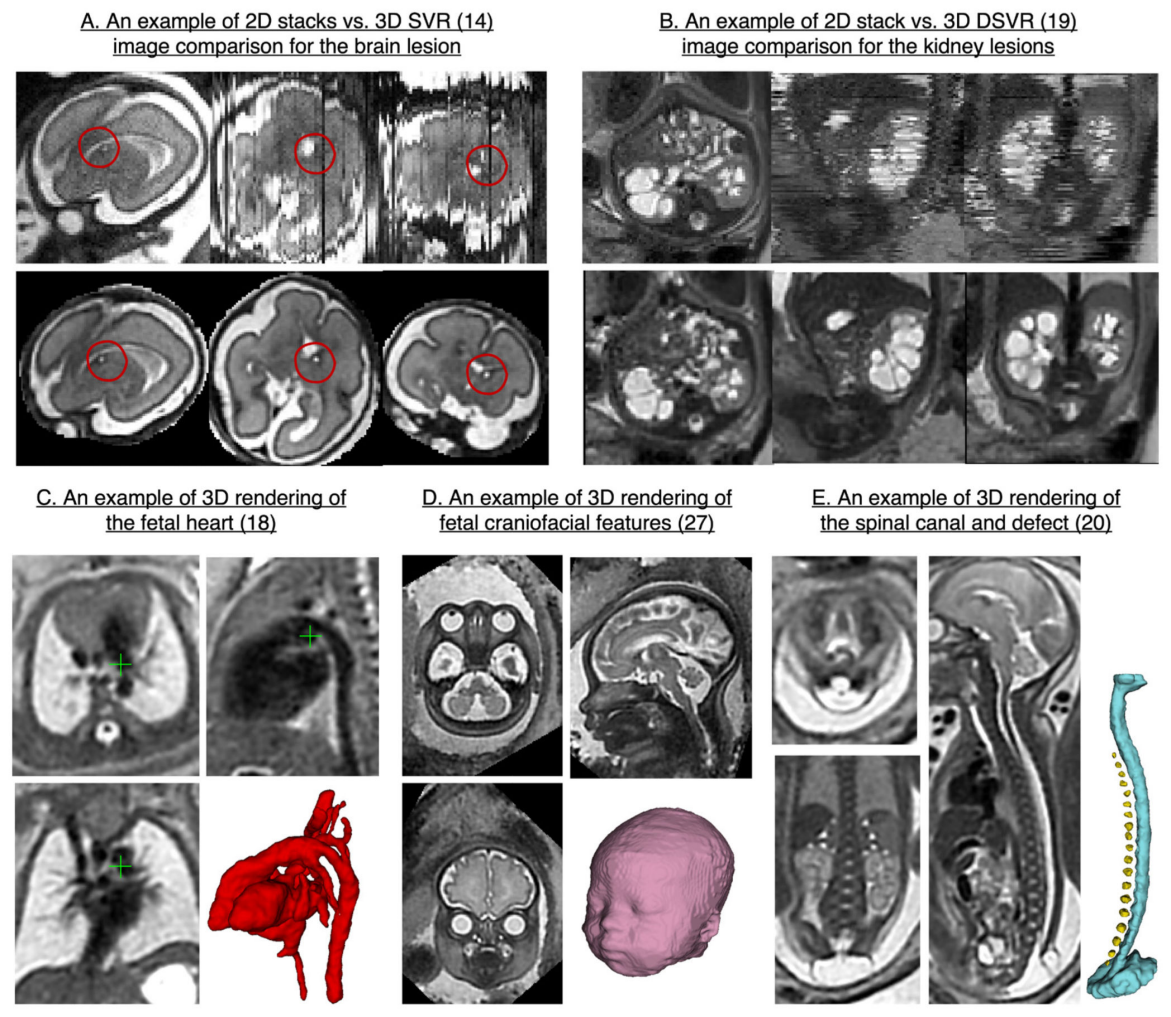
Visualisation of 3D reconstructed images for diagnostic purposes. The examples are based on the MRI datas acquired at St.Thomas’ Hospital, London.

3D foetal MRI also allows better visualisation of more subtle anomalies and fine anatomy structures as it offers continuous volumetric representation of the anatomy. Figure 6a-b show comparison between the original 2D motion-corrupted stacks and 3D SVR and DSVR images for brain and kidney lesions. The improved diagnostic information content was quantitatively confirmed in several studies. For example, 3D reconstruction of the heart (Figure 6c) has increased the number of visualised cardiac vascular structures in 86 foetuses from 53 to 97% compared to acquired motion-corrupted stacks of 2D MRI slices.^18^ Volumetric reconstruction improved diagnostic confidence in diagnosis in 90% of congenital cardiovascular cases.

Among other promising applications is diagnosis of craniofacial abnormalities based on rendering the foetal face in 3D (Figure 6d).^27^ 3D rendering can also potentially aid foetal and neonatal surgical repair planning for body^20^ (Figure 6e) and head and neck^28^ anomalies. However, more clinical studies are needed to identify and confirm improvements in diagnosis and treatment planning for various pathologies. Furthermore, there has been no reported evaluation studies of the conditions when low reconstruction quality might lead to decreased diagnostic content.

### Quantification

**Figure 7:**
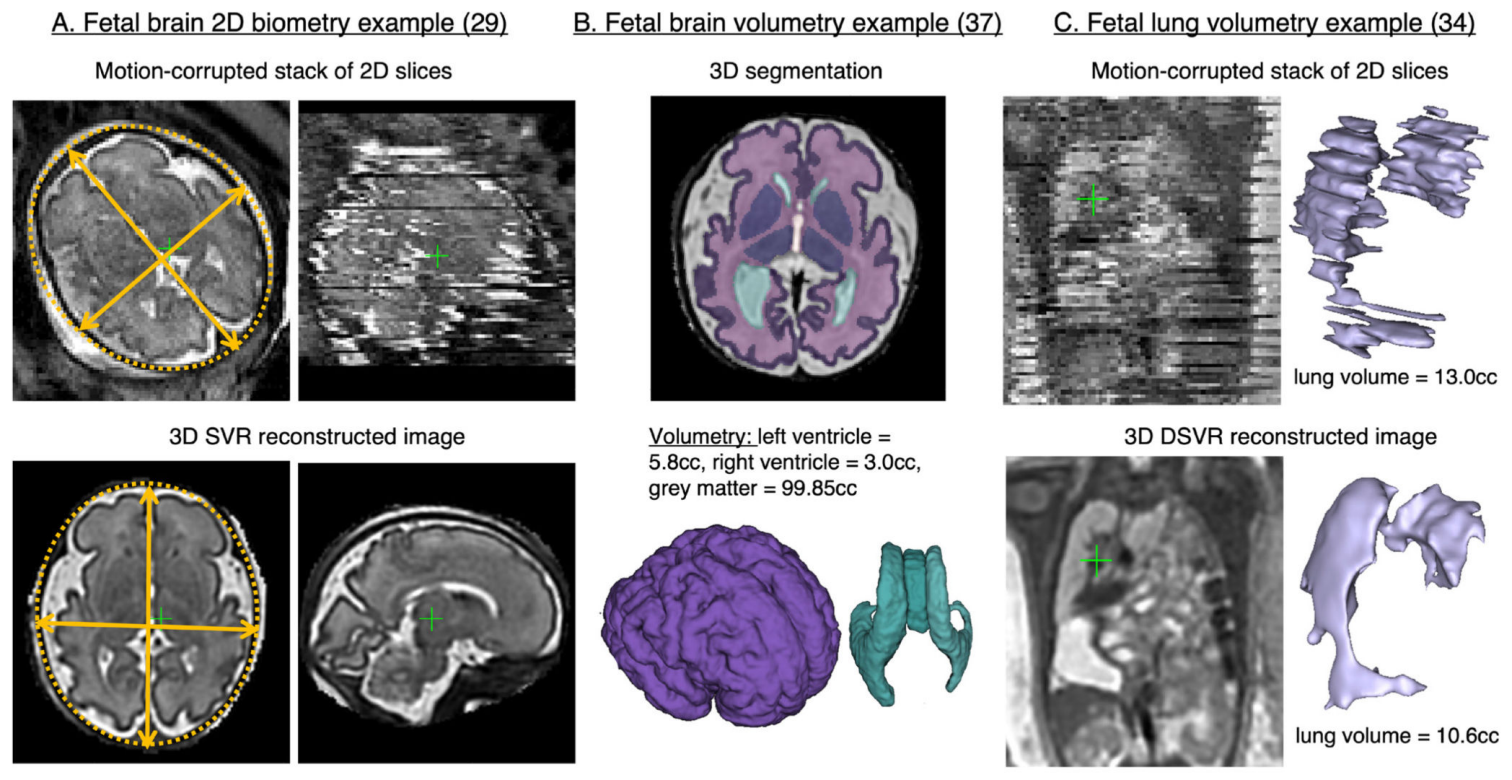
Examples of existing quantitative applications for 3D MRI reconstructed images. The examples are based on the MRI datasets acquired at St.Thomas’ Hospital, London. SVR, slice-to-volume registration.

3D foetal MRI is also beneficial in terms of the improved accuracy of biometric and volumetric measurements. Acquired 2D planes are often inaccurate due to foetal motion during the scan (*e.g*. Figure 7a) which might lead to unreliable 2D biometry. As an alternative, 3D images can be reoriented to any plane. The results of^29^ and^30^ studies confirmed that 2D foetal brain biometric measurements from 2D acquisitions (with precise radiological acquired planes) and 3D reconstructions are highly correlated without significant difference.

Volumetric continuity of reconstructed images allows accurate delineation of structures in 3D and potentially consistent volumetry of organs and lesions. In comparison, the conventional 2D slice-wise segmentations performed on the original stacks^31^ are sensitive to motion (Figure 7c). Lung volumetry is used in prediction of post-natal lung function in congenital diaphragmatic hernia^32^ and may play an important role in patient selection for the new foetal surgical interventions.^33,34^ reported that for the cases with minor to average motion, the volumetric measurements of foetal lungs from 2D stacks of slices and DSVR corrected motion corrected 3D images^19^ are highly consistent. Decreased volumetric measurements of foetal thymus, lungs and brain have also been linked to high risk of premature delivery.^35–37^ Figure 7b shows the format of 3D volumetric brain segmentation outputs used in.^37^

The currently clinically accepted 2D foetal MRI and ultrasound-derived volumetry is intrinsically prone to errors and cannot be considered to be the precise ground truth. This makes validation of 3D MRI-derived measurements for severe motion datasets a challenging task. Yet, before 3D MRI volumetry can be used in clinical practice, it is necessary to establish that there is a consistent correlation with conventional 2D foetal MRI and ultrasound.

### Translation into clinical practice

Currently, all SVR-based reconstruction tools are still in the research stage and not commercially available. In addition to the extensive evaluation and approval by regulatory agencies, translation into clinical practice depends on a set of practical aspects. This includes consistency of reconstruction quality, functioning user interface, compliance with data protection regulations, reasonable processing time and minimal manual input.

### Selection of the optimal SVR reconstruction method

During the past decade, there have been many proposed SVR implementations that addressed different components of the algorithm. However, the core of all methods relies on the same theory (Figure 3a). There are three main existing modern toolboxes that provide functionality for foetal brain reconstruction. They are used on a regular basis at different clinical research facilities and can be easily installed on a regular workstation:

- MialSRTK: University of Lausanne, Switzerland, based on^15,38^
- NiftyMIC: University College London, UK, based on^23,39^
- SVRTK: King’s College London, UK, based on^14,40^

**Figure 8:**
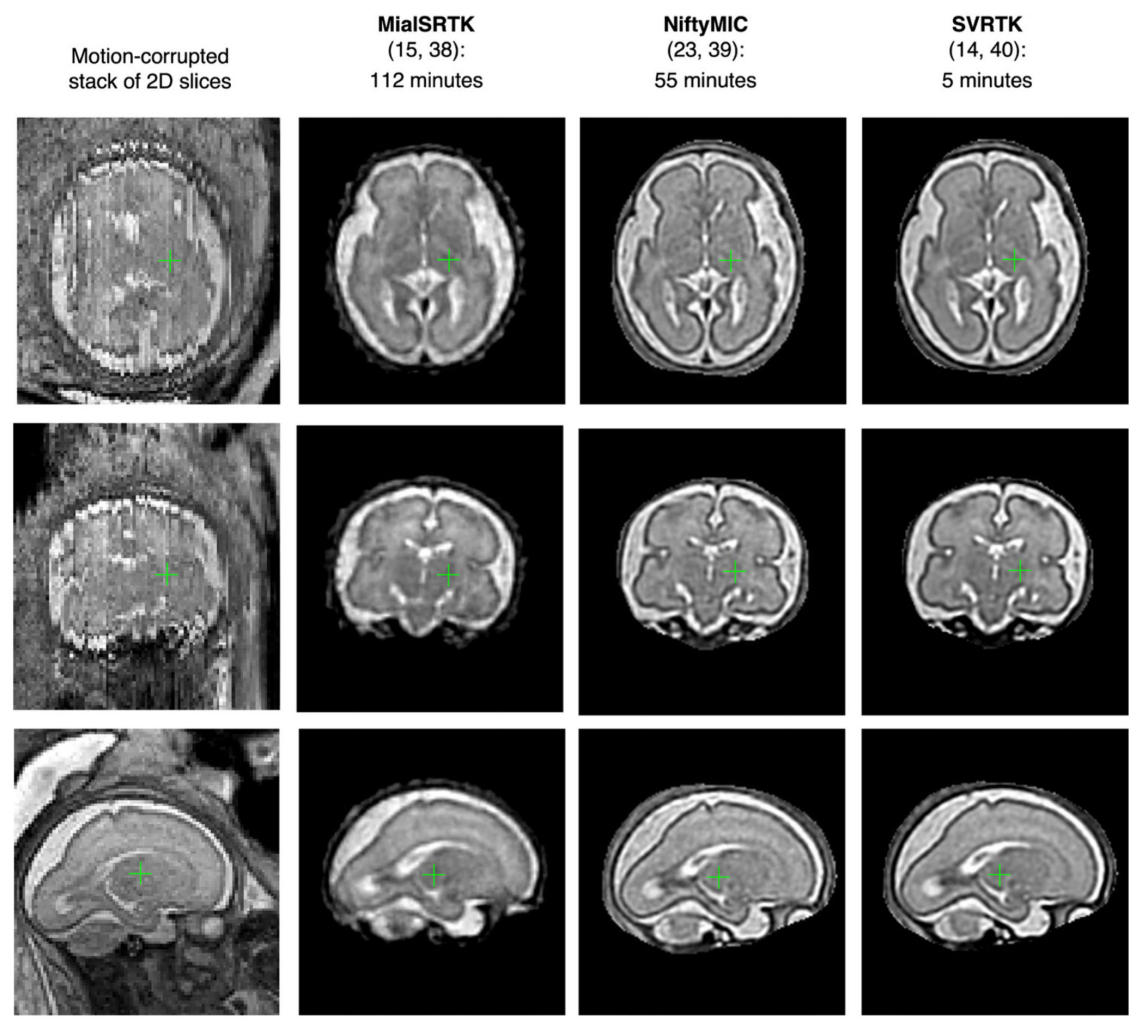
Comparison of different SVR reconstruction toolboxes for foetal brain MRI. SVR, slice-to-volume registration.

During the past decade, there have been many works on comparison of various aspects of different SVR methods (*e.g*., ^41^). In general, these methods produce similar results for the foetal brain anatomy and require manually created 3D brain mask as an input. The choice of a toolbox would be primarily dictated by the preferences towards a specific method and reconstruction time limits. To confirm this finding, Figure 8 shows example comparison of these methods for a minor motion normal 28 weeks GA dataset with six stacks (1.5 T SSTSE protocol, Figure 2). The sharpness of image features depends on the specifics of the implemented methods.

The expected processing time plays a critical role in the integration of the methods into clinical practice. To our knowledge, there has been no reported comparison of these methods in terms of time. In general, the processing time depends on both the hardware and implementation of the method. In this example performed on a regular CPU-only workstation (via the dedicated toolbox CPU Dockers), the reconstruction time for remialSRTK, NiftyMIC and SVRTK was 112, 55 and 5 min, correspondingly. The SVRTK reconstruction was significantly faster because it is based on parallelisation and fast *C* ++ image processing library unlike Python-based NiftyMIC. The processing speed is directly not related to the output image quality. In addition, NiftyMIC^39^ and SVRTK^40^ toolboxes provide direct options for automated brain masking and reorientation to the standard space for fully automated reconstruction that do not require any manual input. For this dataset, the automated brain masking (on CPU Docker) in NiftyMIC increased the total processing time by 126min, while both brain masking and reorientation took only 4min in SVRTK.

In addition, SVRTK package provides functionality for manual and automated DSVR reconstruction of the foetal body.^19^

### User interface, hardware requirements and installation

These three toolboxes are distributed via Docker containers which requires only minimal installation steps and the reconstruction process can be directly launched via command line. However, integration of SVR tools into a hospital setting requires a dedicated user interface for clinicians without a technical background. Compliance with data protection regulations also means that all reconstructions should be performed on a secure server with protected data storage and transfer. The total processing time (Figure 8) is another key aspect.

**Figure 9:**
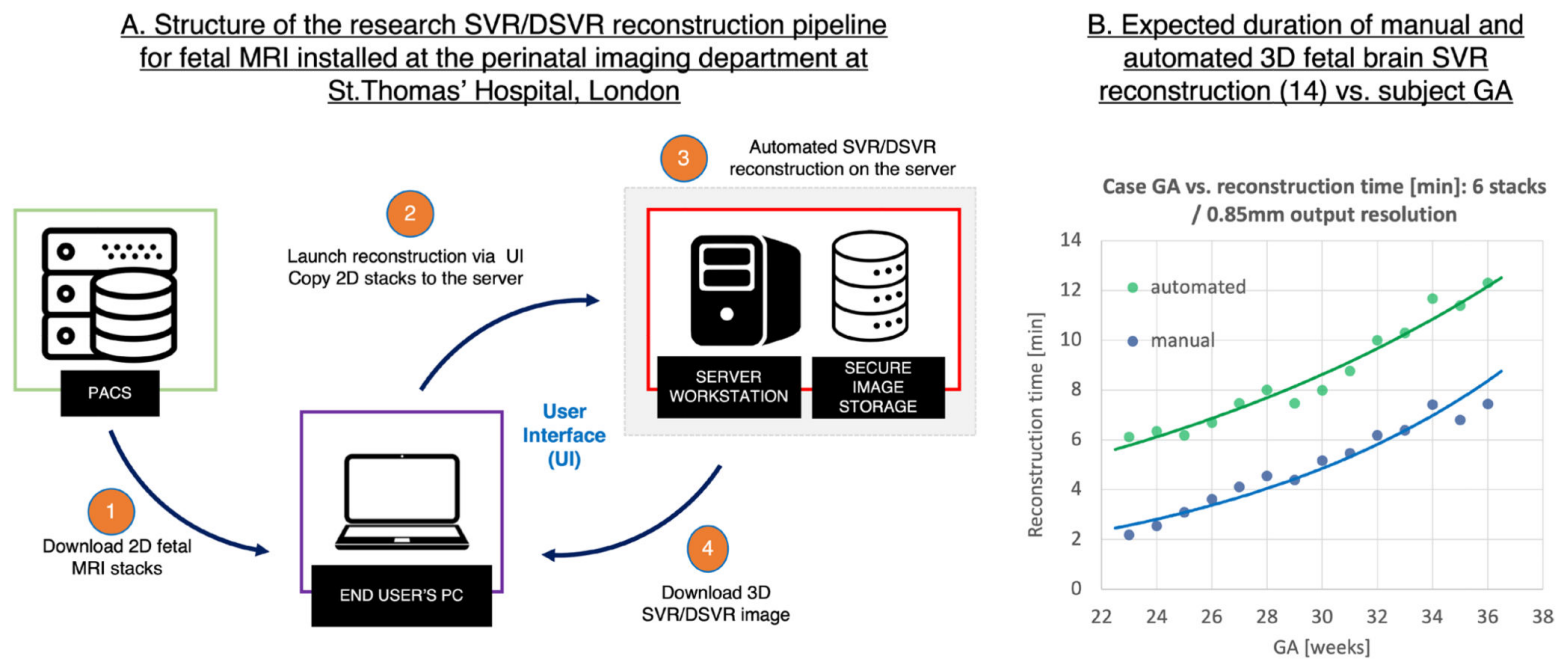
An example of integration of 3D SVR/DSVR reconstruction for foetal MRI into clinical settings (Perinatal Imaging Department at St.Thomas’ Hospital, London). SVR, slice-to-volume registration.

Figure 9a shows the SVR/DSVR reconstruction pipeline designed for the research purposes at St.Thomas’ Hospital, London. 3D reconstruction of the foetal brain^14^ and body^18,19^ can be performed directly by clinicians. In this pipeline, a user launches a script that copies input 2D *T2*W SSTSE foetal MRI stacks to the server and starts the SVR or DSVR reconstruction process. The output reconstructed 3D image is automatically stored on the dedicated secure local storage database for visualisation and diagnosis. It requires only minimal user input and a mask for one of the stacks, if the automated masking^42^ was not selected.

An alternative future direction would be to use vendor-specific connection interfaces between the scanner console and remote workstations (such as the Philips Research Imaging Development Environment) that would allow SVR reconstruction to be launched directly during scanning and storage of output 3D images as a part of the patient datasets.

Figure 9b shows the expected time for SVR reconstruction^14^ of the foetal brain on a regular workstation using six stacks (1.5 T SSTSE protocol, Figure 2). The reconstruction time increases with the GA due to the larger brain size. Notably, the automated SVR steps take ~4 min in addition to the main SVR reconstruction. The reconstruction will be faster for datasets with less stacks or thick slices because of the smaller total number of slices.

### Input data requirements, image quality, limitations and validation

**Figure 10:**
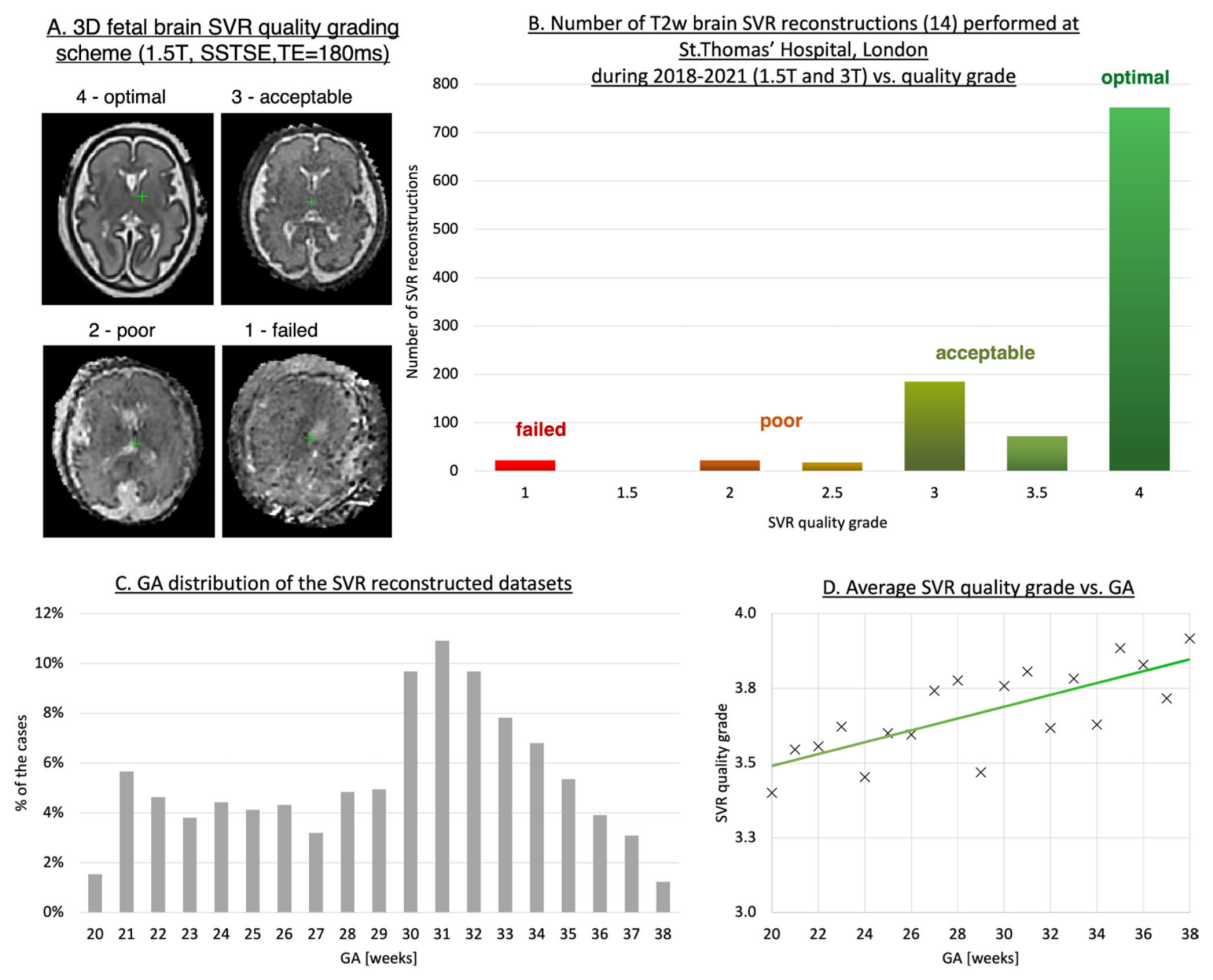
An example of qualitative assessment of the classical 3D SVR^14^ reconstruction for foetal brain MRI on a large (-1000 cases) single-centre cohort. The datasets were acquired and processed at St.Thomas’ Hospital, London. GA, gestational age; SVR, slice-to-volume registration.

As part of ongoing research projects, since 2018, SVR reconstructions have been routinely performed for all foetal brain MRI datasets acquired at the Perinatal Imaging Department at St.Thomas’ Hospital, London. Figure 10b shows distribution of 3D foetal brain reconstructed image quality of 1070 reconstructions from 2018 to 2021 period for both clinical reporting and research projects. The datasets were processed using the classical SVR method^14^ with either manual or automated masking. The acquisitions were performed on both a 1.5T Philips Ingenia scanner and a 3T Philips Achieva scanner using torso receiver array coils and *T2W* SSTSE protocols (Figure 2) with 5–9 stacks per dataset.

The grading scheme shown in Figure 10a has a range of 1–4 with four corresponding to high quality images. The causes of Grades 2–3 (slight or severe SNR and contrast loss) are primarily related to low quality of the original 2D stack. The failed reconstruction with grades < 1.5 were mainly caused by large rotations (>60°). The cases with grades ≥ 2.5 are considered to be acceptable for clinical interpretation and ~96% of all SVR reconstructions were successful. The GA range of the cases varied between 20 and 38 weeks (Figure 10c) with the average quality grade being higher for late GA datasets (Figure 10d).

Furthermore, since 2016, more than 400 SVR/DSVR reconstructions of the foetal thorax (primarily aortic arch anomalies) have been performed for *T2W* black blood datasets as a part of the clinical foetal CMR service at St.Thomas’ Hospital, London.^18^ More recently, the DSVR method has also been routinely used for foetal body MRI datasets^20^ with sufficiently high reporting quality grading (>90%) rates.^19^

However, even for cases with high reconstruction quality, clinical reporting should currently include inspection of the original 2D stacks, until clinical studies confirm reliability of 3D reconstructed MRI for diagnosis. This will prevent any potential false findings or missed information due to artefacts propagated from the original slices or averaging.

The current limitations of 3D SVR/DSVR include poor contrast due to severe motion or extreme intensity artefacts (Figure 10a). In addition, averaging of dynamic organ motion (*e.g*. swallowing) may limit the diagnosis of abnormalities of gastrointestinal tract, such as oesophageal atresia and bowel obstruction. None of the existing implementations is currently suitable for reconstruction of the limbs due to the extremely large motion trajectories. Furthermore, the automated masking and reorientation steps can fail in cases of low input image quality, extreme anomalies or multiple gestation pregnancies.

In general, large-scale integration of SVR-based motion correction tools into clinical practice will require further optimisation of the existing tools and user interface, extensive validation and formalisation of the processing and diagnosis protocols. Although this should not lead to extreme cost implications to clinical sites, the additional research will require a significant research and software development input as well as multicentre validation studies.

### Current research and future clinical practice

Current 3D MRI research projects primarily focus on automation of organ segmentation in SVR-reconstructed images, definition of the normal and abnormal development models (atlases, growth charts) and optimisation of SVR-based methods for advanced MRI acquisition protocols.

### 3D MRI atlases of foetal development, volumetry growth charts

**Figure 11:**
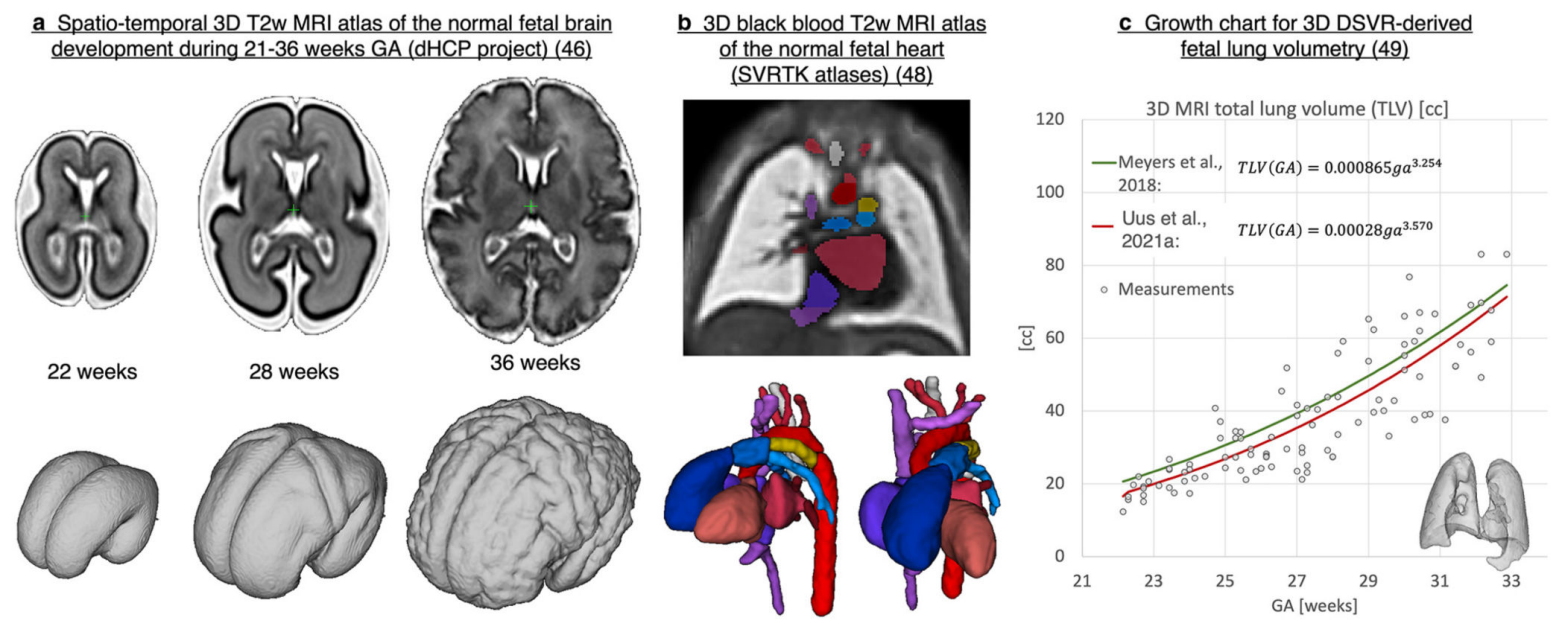
Examples of 3D MRI-derived foetal development models. GA, gestational age; SVR, slice-to-volume registration.

There are several released 3D MRI atlases defining normal^43,44^ and abnormal^45^ foetal brain development. They consist of weekly templates generated by averaging of registered 3D SVR-reconstructed images. For example, Figure 11a shows a 3D T2W spatio-temporal atlas of the foetal brain covering 22 to 36 weeks range^46^ generated from the Developing Human Connectome Project (dHCP)^47^ datasets. There are also released 3D MRI atlases of the foetal heart^48^ (Figure 11b) and lungs.^49^ In addition to educational purposes, 3D MRI atlases are widely employed for automated registration-guided segmentation based on label propagation.^41,45,49^

3D organ segmentations derived from 3D MRl have been employed for modelling of growth charts for normal and abnormal development of the foetal brain^37,50^ and other organs such as lungs^35,49^ (Figure 11c). However, there is a general lack of standardised growth models for foetal organ volumetry due to the absence of systematic segmentation protocols and variation in segmentation methods.

### Artificial intelligence for automated 3D foetal organ segmentation

The majority of the proposed modern methods for automated segmentation of 3D MRI images are based on AI methods, specifically 3D convolutional neutral networks. The recent FeTA challenge^41^ demonstrated that AI provides robust performance for foetal brain tissue segmentation in *T2W* 3D SVR images. AI solutions can operate for both normal^51,52^ and abnormal anatomy.^53^ the current challenges are the lack of standardised manual ground truth 3D segmentation and harmonisation with respect to different acquisition protocols.

### Advanced SVR-based reconstruction methods for foetal MRI

**Figure 12:**
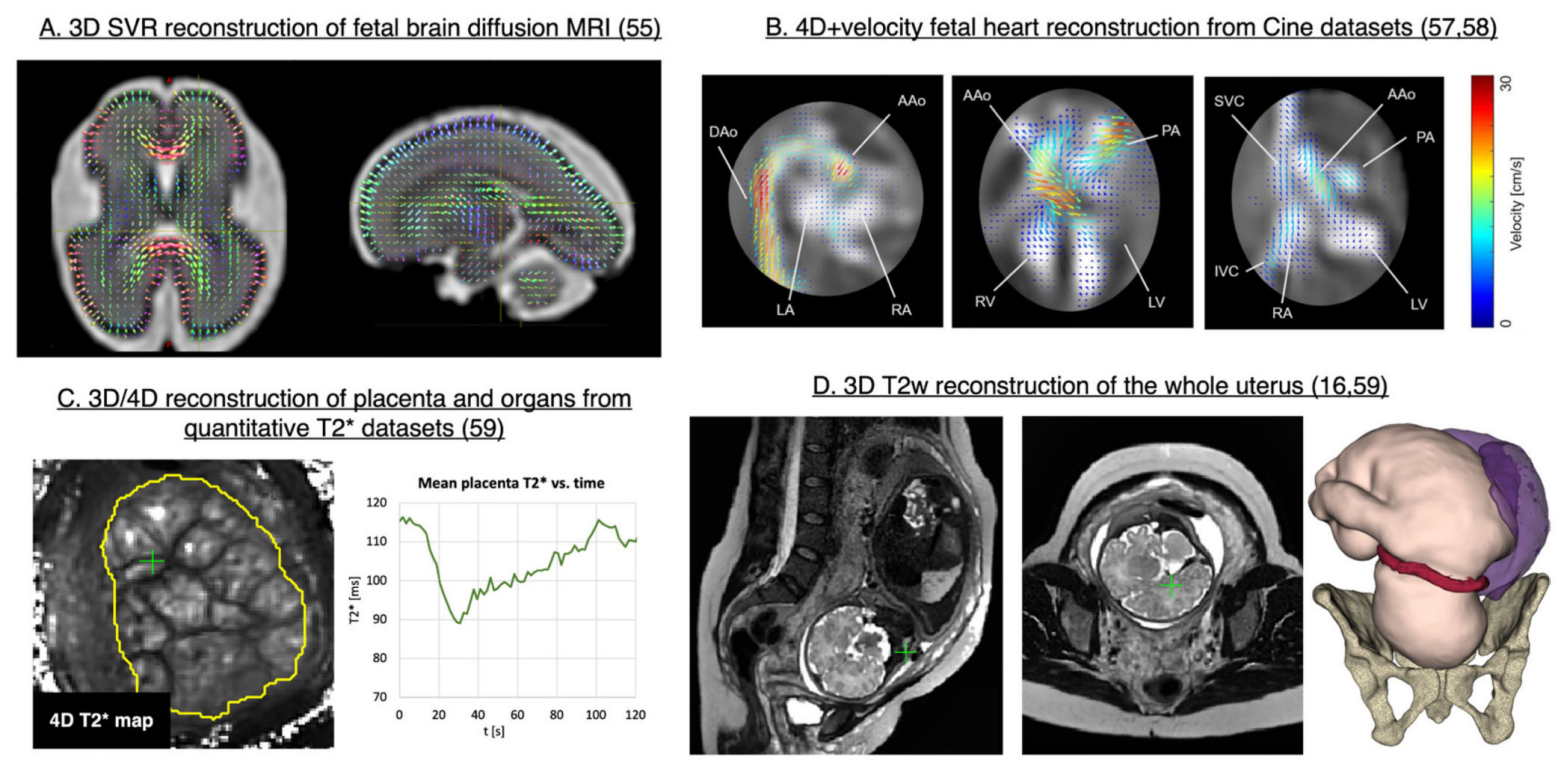
Examples of SVR-based solutions for advanced foetal MRI acquisition methods. SVR, slice-to-volume registration.

In addition to the 3D static motion correction tools for *T2*W foetal brain and body imaging, there are a number of SVR-based solutions for advanced MRI acquisition methods. These include foetal brain diffusion MRI,^54–56^ *4D* + velocity Cine heart imaging^57,58^ and quantitative 3D and 4D foetal and placental T2*.^59^ Figure 12a-c show several illustrations from these works. Reconstruction of the whole uterus^16,19,59^ (Figure 12d) also provides information on the extrafoetal structures, such as the umbilical cord, placenta and cervix and the amniotic fluid.

## Conclusion

Foetal MRI is a complementary imaging method to ultrasound that provides additional information for antenatal diagnosis of lesions and anomalies. However, even with the advances in fast acquisition sequences, foetal MRI datasets are intrinsically corrupted by motion that limits volumetric information in stacks of 2D slices.

Motion correction methods based on SVR provide the means to perform 3D reconstruction of isotropic high-resolution static images of the foetal brain and body. Reconstructed 3D MRI images can be reoriented in any plane and visualised via volume rendering thus allowing full volumetric representation of the foetal anatomy. 3D foetal MRI can improve diagnostic accuracy and confidence, and it enables true 3D segmentation for quantification of organ volumetry, which is not possible when using conventional, error-prone 2D slice-wise imaging protocols. 3D SVR/DSVR methods also have the potential to decrease acquisition times by removing the need for manual readjustment of acquisition planes to the standard radiological views.

Since structural *T2*-weighted imaging of the foetal brain and body is the foundation of foetal MRI, 3D SVR-based static motion correction and automated processing have been already extensively investigated during the past 15 years. Research studies utilising SVR methods to produce motion-corrected volumes have shown much potential and 3D foetal MRI is gradually progressing towards clinical validation. For routine integration into clinical settings, extensive evaluation and standardisation of the different components of the SVR reconstruction methods and optimisation of the existing software solutions is required.

## Supplementary Material

Figure S1

Figure S2

Figure S3

Figure S4

Video S1

Video S2

Video S3
