# Supplementary figures and images for: Retrospective motion correction in foetal MRI for clinical applications: existing methods, applications and integration into clinical practice
Source: Br J Radiol. 2022 Jul 25;96(1147):20220071. doi: 10.1259/bjr.20220071 (PMC7614695; doi:10.1259/bjr.20220071)

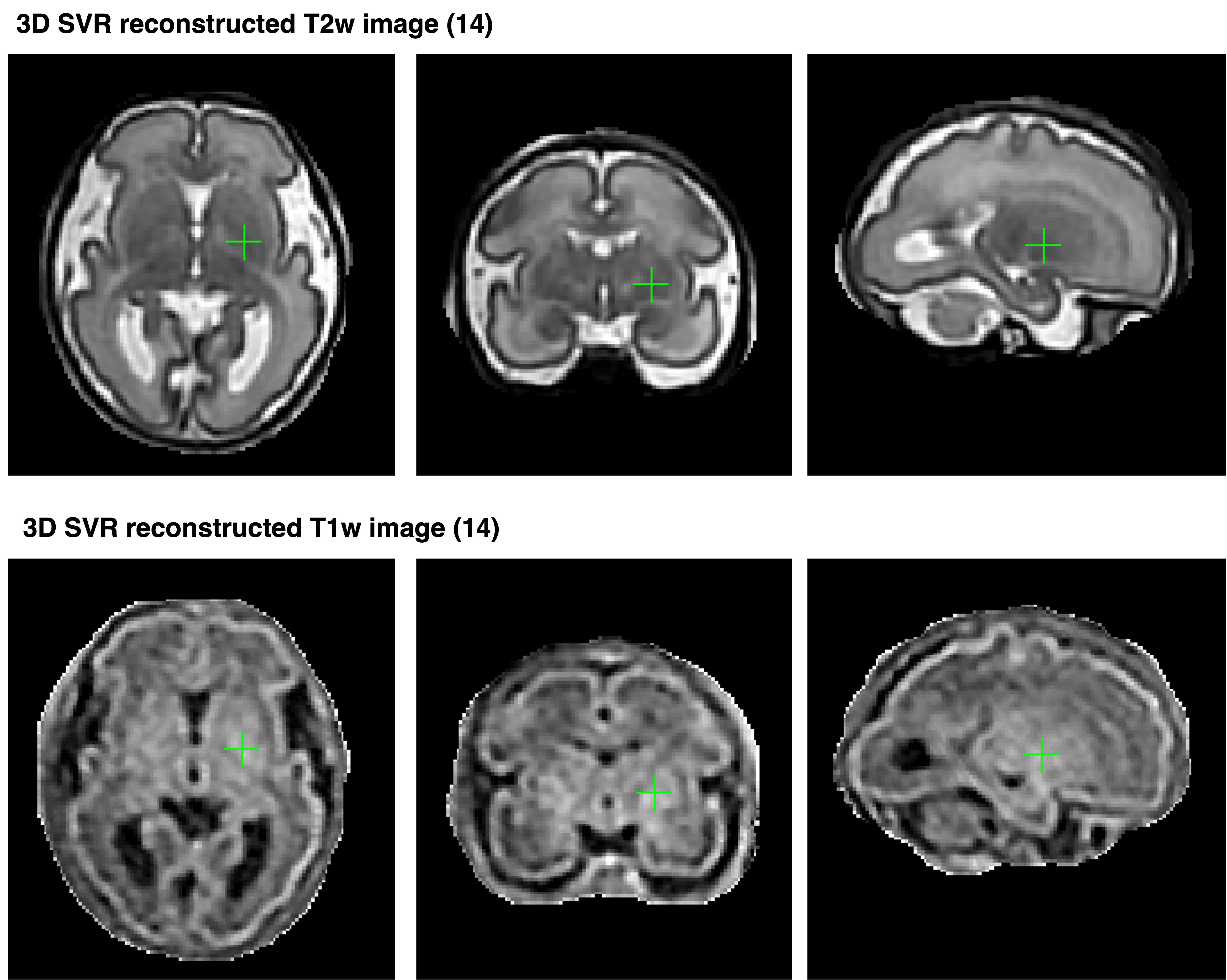

Supplement: Supplementary Figure 1. [file bjr.20220071.suppl-01.jpg]

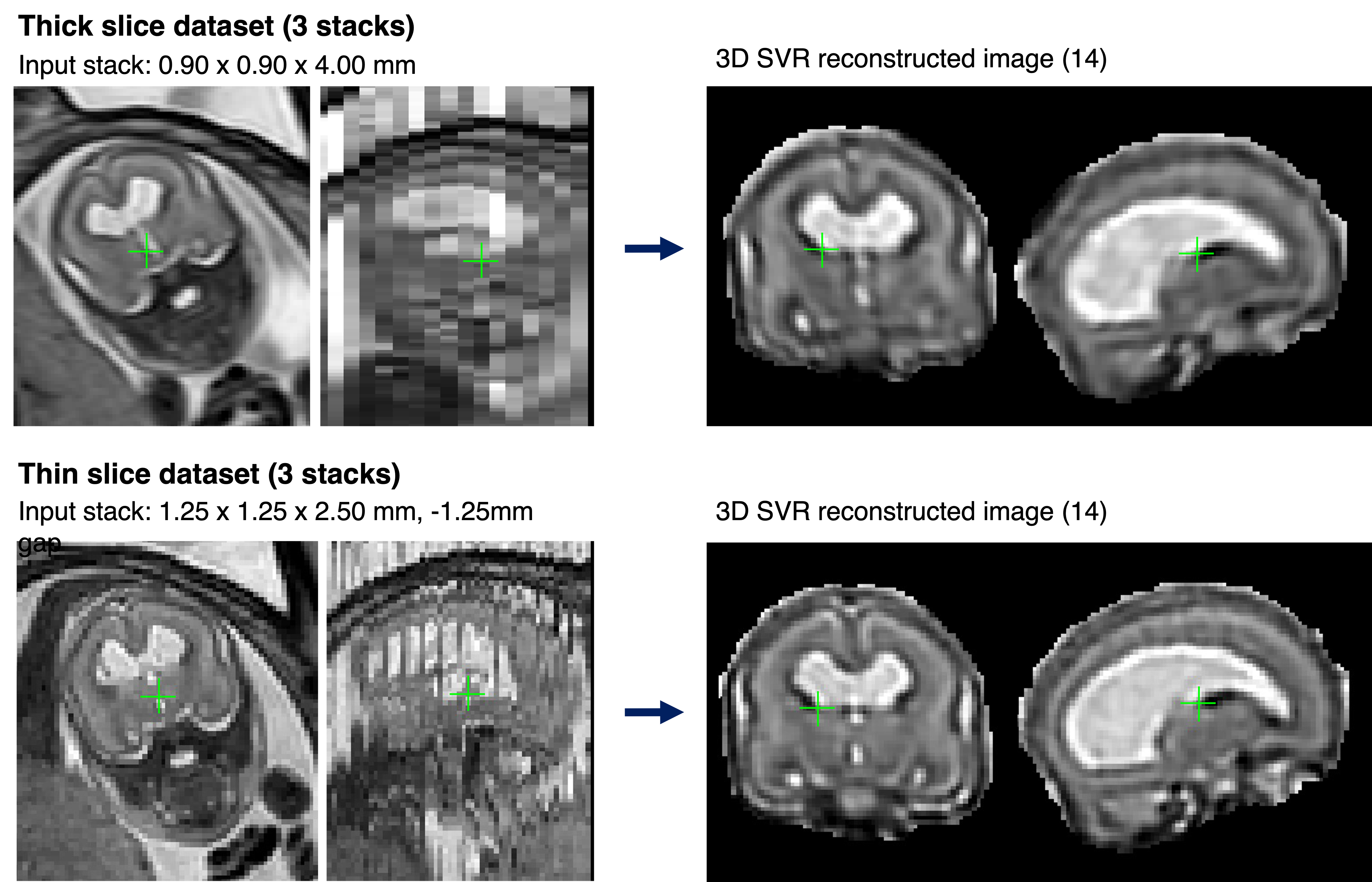

Supplement: Supplementary Figure 2. [file bjr.20220071.suppl-02.jpg]

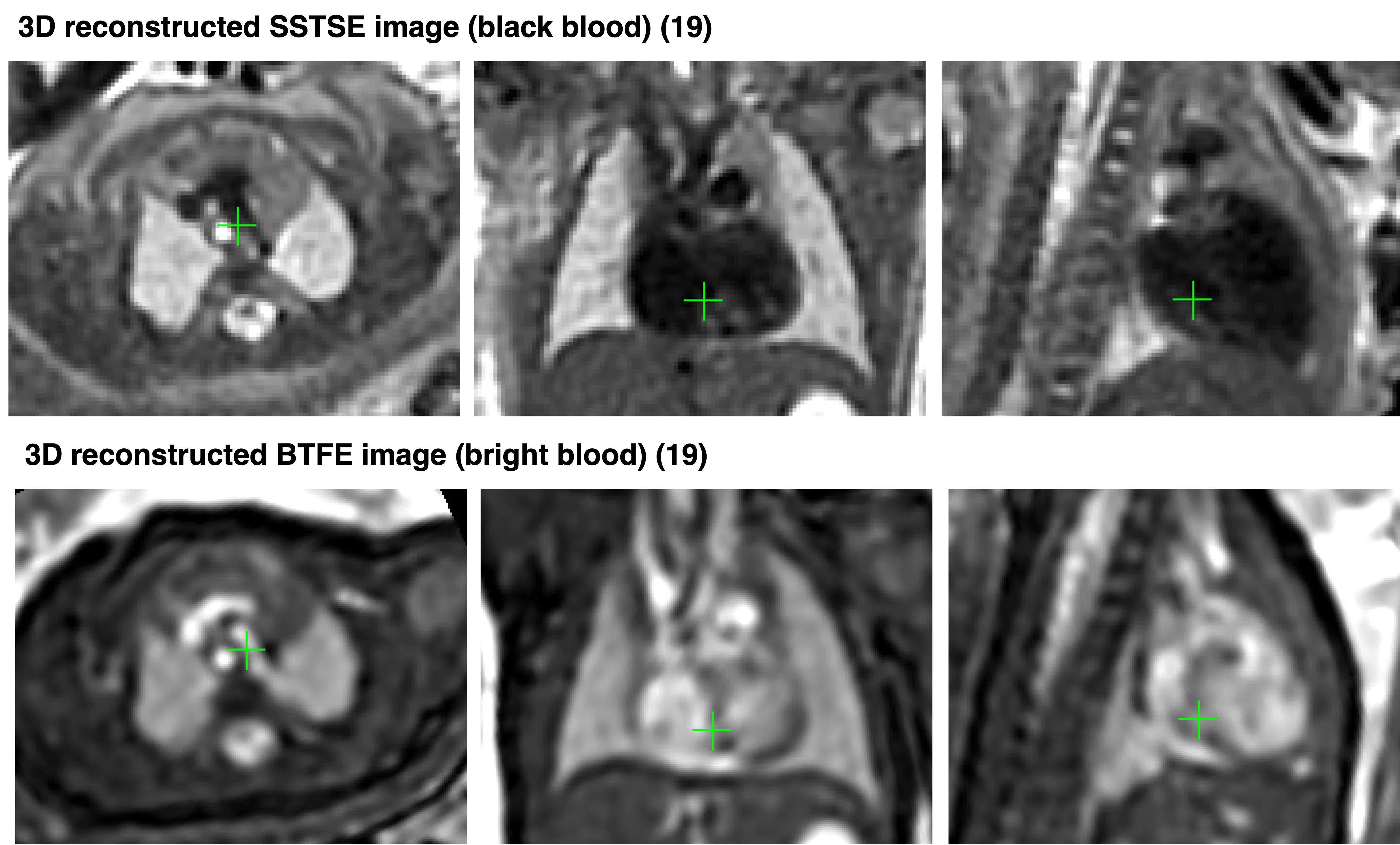

Supplement: Supplementary Figure 3. [file bjr.20220071.suppl-03.jpg]

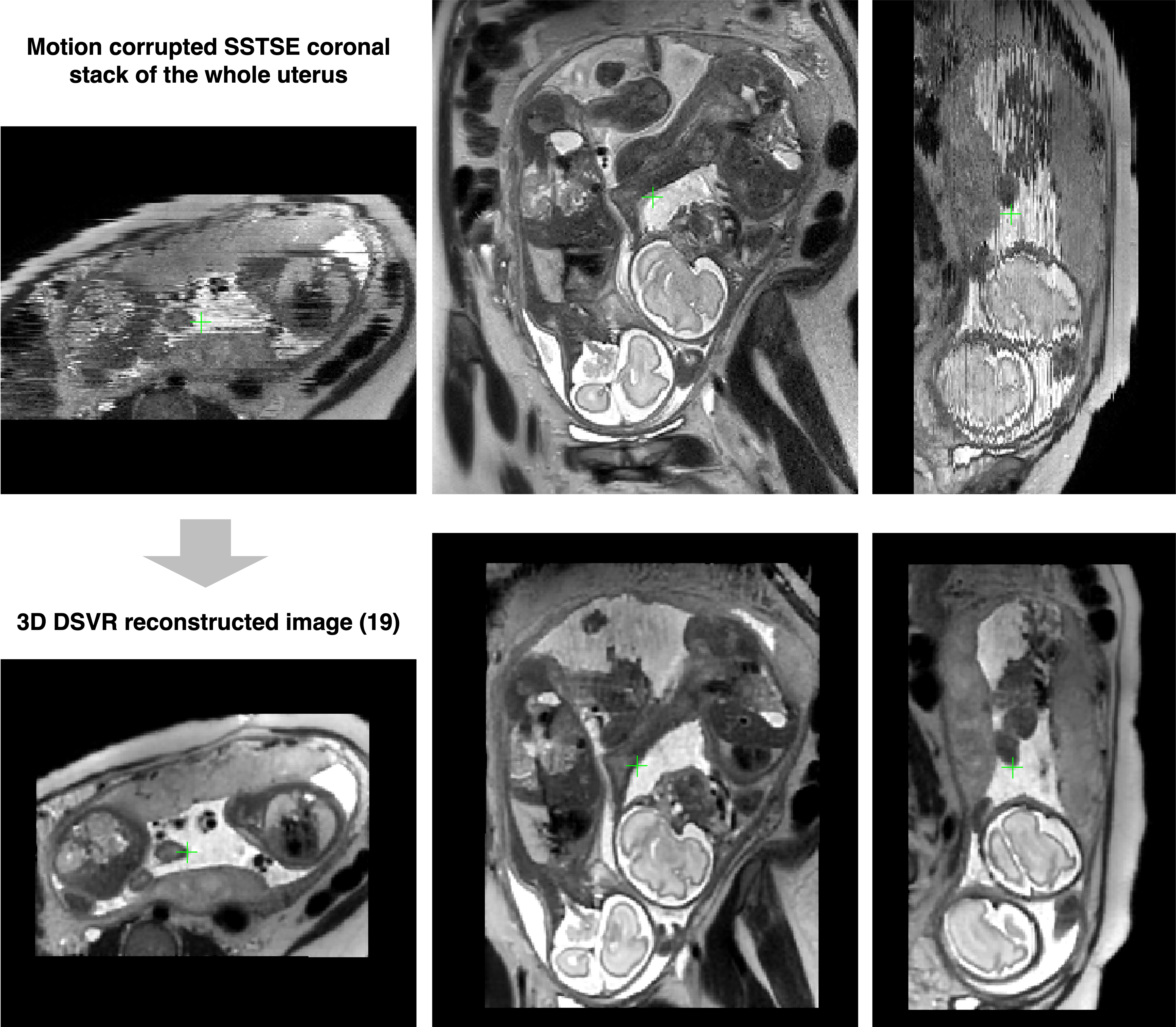

Supplement: Supplementary Figure 4. [file bjr.20220071.suppl-04.jpg]
